# Supplementary material for: Tsetse salivary glycoproteins are modified with paucimannosidic N-glycans, are recognised by C-type lectins and bind to trypanosomes
Source: PLoS Negl Trop Dis. 2021 Feb 2;15(2):e0009071. doi: 10.1371/journal.pntd.0009071 (PMC7880456; doi:10.1371/journal.pntd.0009071)
Supplement: S1 Fig — 10 μg G. morsitans salivary proteins (lanes 1 and 2) and egg albumin (lanes 3 and 4) were incubated overnight with (+) or without (-) PNGase F to cleave N-glycans. Samples were resolved on a 12% SDS-PAGE gel and stained with Colloidal Coomassie Blue (A) or Schiff’s (B) staining. Asterisk indicates PNGase F enzyme. (DOCX) [file pntd.0009071.s001.docx]

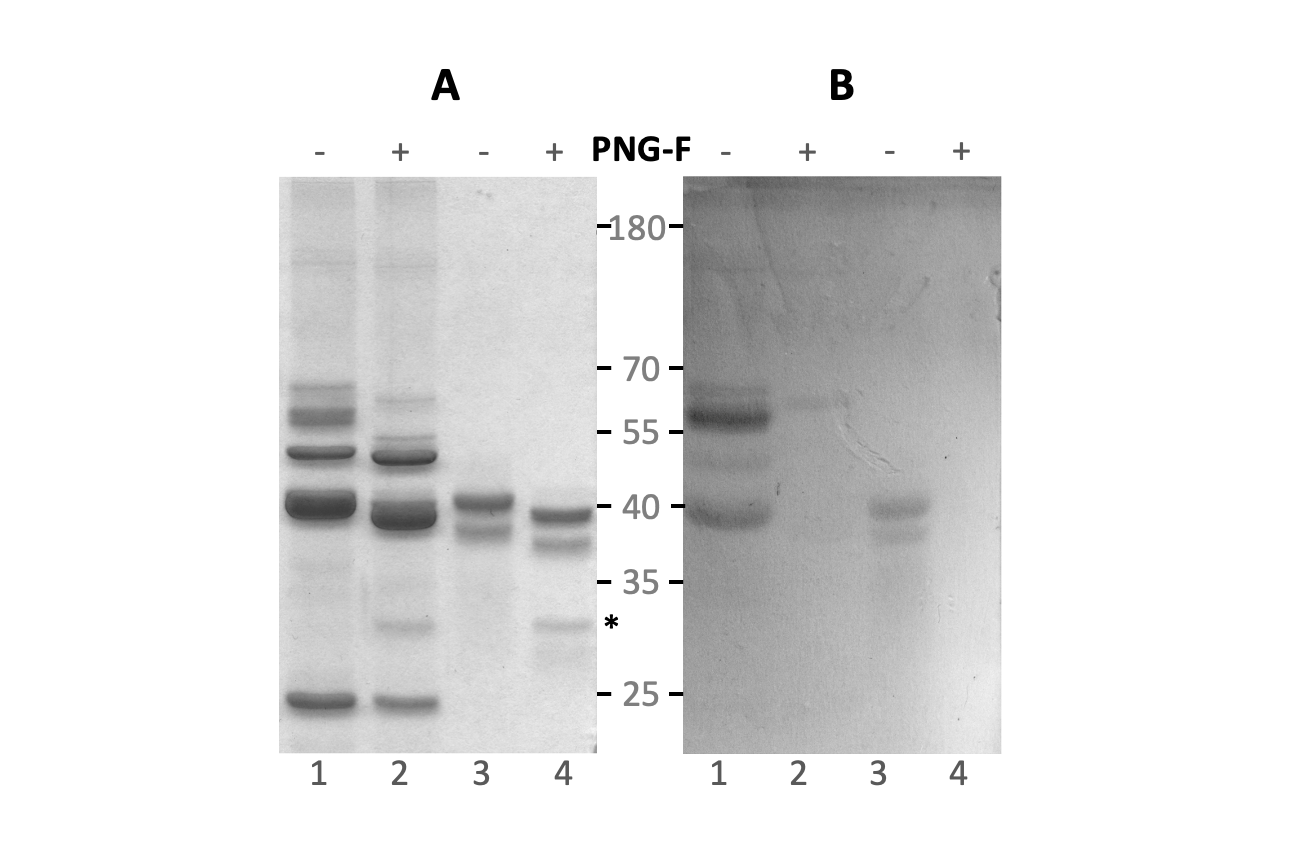


**S1 Fig. Schiff’s staining analysis of *G. morsitans* salivary glycoproteins after enzymatic de-glycosylation.** 10 µg *G. morsitans* salivary proteins (lanes 1 and 2) and egg albumin (lanes 3 and 4) were incubated overnight with (+) or without (-) PNGase F to cleave *N*-glycans. Samples were resolved on a 12 % SDS-PAGE gel and stained with either Colloidal Coomassie Blue (A) or Schiff’s (B) staining. Asterisk indicates PNGase F enzyme.
