# Supplementary material for: Tsetse salivary glycoproteins are modified with paucimannosidic N-glycans, are recognised by C-type lectins and bind to trypanosomes
Source: PLoS Negl Trop Dis. 2021 Feb 2;15(2):e0009071. doi: 10.1371/journal.pntd.0009071 (PMC7880456; doi:10.1371/journal.pntd.0009071)
Supplement: S2 Fig — 10 μg G. morsitans salivary proteins (lanes 1 and 2) and egg albumin (lanes 3 and 4) were incubated overnight with (+) or without (-) Endo H. Samples were resolved on a 12% SDS-PAGE gel and Coomassie stained. There was a notable shift in migration in 4 bands (1–4) after deglycosylation. These bands were excised, trypsinised and identified by mass spectrometry. 1, 5’ Nucleotidase; 2, TSGF 2/Adenosine deaminase; 3, TSGF 1; 4, Tsal 1/2. Asterisk indicates Endo H. (DOCX) [file pntd.0009071.s002.docx]

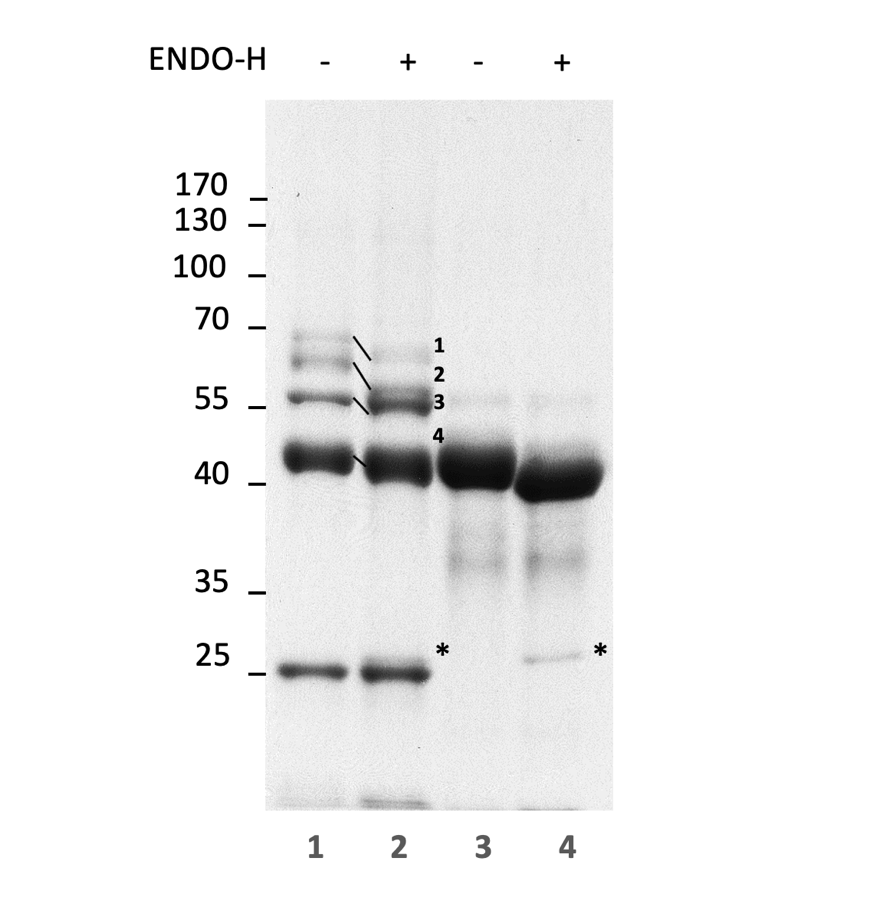


**S2 Fig. Endo H** **cleavage of *G. morsitans* salivary glycoproteins.** 10 µg *G. morsitans* salivary proteins (lanes 1 and 2) and egg albumin (lanes 3 and 4) were incubated overnight with (+) or without (-) Endo H. Samples were resolved on a 12 % SDS-PAGE gel and Coomassie stained. There was a notable shift in migration in 4 bands (1-4) after deglycosylation. These bands were excised, trypsinised and identified by mass spectrometry. 1, 5’ Nucleotidase; 2, TSGF 2/Adenosine deaminase; 3, TSGF 1; 4, Tsal 1/2. Asterisk indicates Endo H.
