# Supplementary material for: Tsetse salivary glycoproteins are modified with paucimannosidic N-glycans, are recognised by C-type lectins and bind to trypanosomes
Source: PLoS Negl Trop Dis. 2021 Feb 2;15(2):e0009071. doi: 10.1371/journal.pntd.0009071 (PMC7880456; doi:10.1371/journal.pntd.0009071)
Supplement: S3 Fig — (A) N-glycans released by PNGase A, (B) N-glycans released by PNGase F, (C) N-glycans released by PNGase A after deglycosylation with PNGase F. Green circle, mannose; blue square, N-acetylglucosamine; red triangle, fucose. Peaks labelled with an asterisk refer to buffer contaminants. (DOCX) [file pntd.0009071.s003.docx]

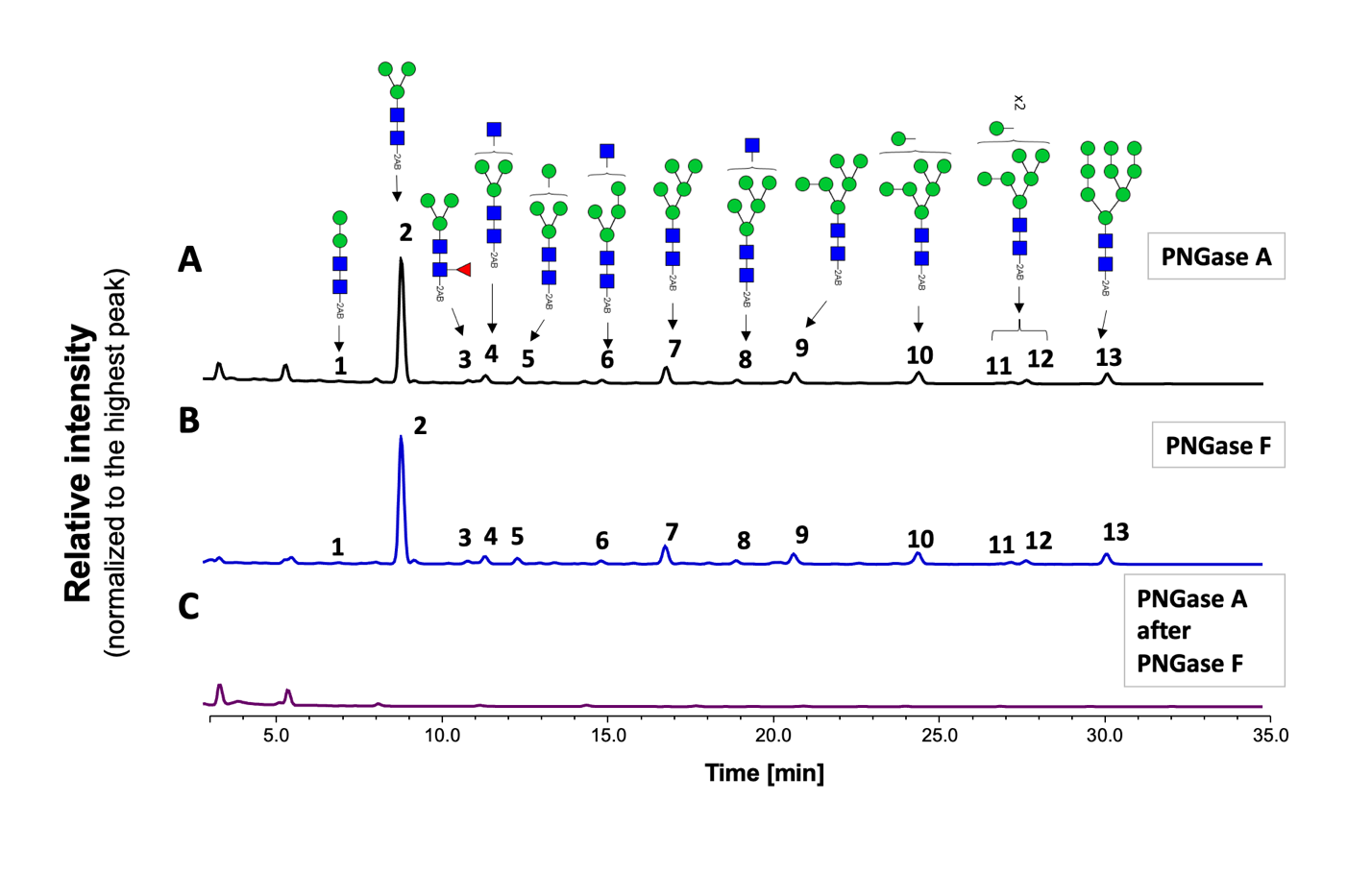


**S3 Fig**. **HILIC-UHPLC chromatograms of 2-AB labelled *N*-glycans released enzymatically from teneral fly saliva**. (A) *N*-glycans released by PNGase A, (B) *N*-glycans released by PNGase F, (C) *N*-glycans released by PNGase A after deglycosylation with PNGase F. Green circle, mannose; blue square, *N*-acetylglucosamine; red triangle, fucose. Peaks labelled with an asterisk refer to buffer contaminants.
