# Supplementary material for: Tsetse salivary glycoproteins are modified with paucimannosidic N-glycans, are recognised by C-type lectins and bind to trypanosomes
Source: PLoS Negl Trop Dis. 2021 Feb 2;15(2):e0009071. doi: 10.1371/journal.pntd.0009071 (PMC7880456; doi:10.1371/journal.pntd.0009071)
Supplement: S4 Fig — Spectra correspond to (A) m/z 1130.49 (Man3GlcNAc2-Proc), (B) m/z 1276.52 (Man3GlcNAc2Fuc-Proc), (C) m/z 1333.57 (Man3GlcNAc3-Proc). Green circle, mannose; blue square, N-Acetylglucosamine; red triangle, fucose; Proc, procainamide. (DOCX) [file pntd.0009071.s004.docx]

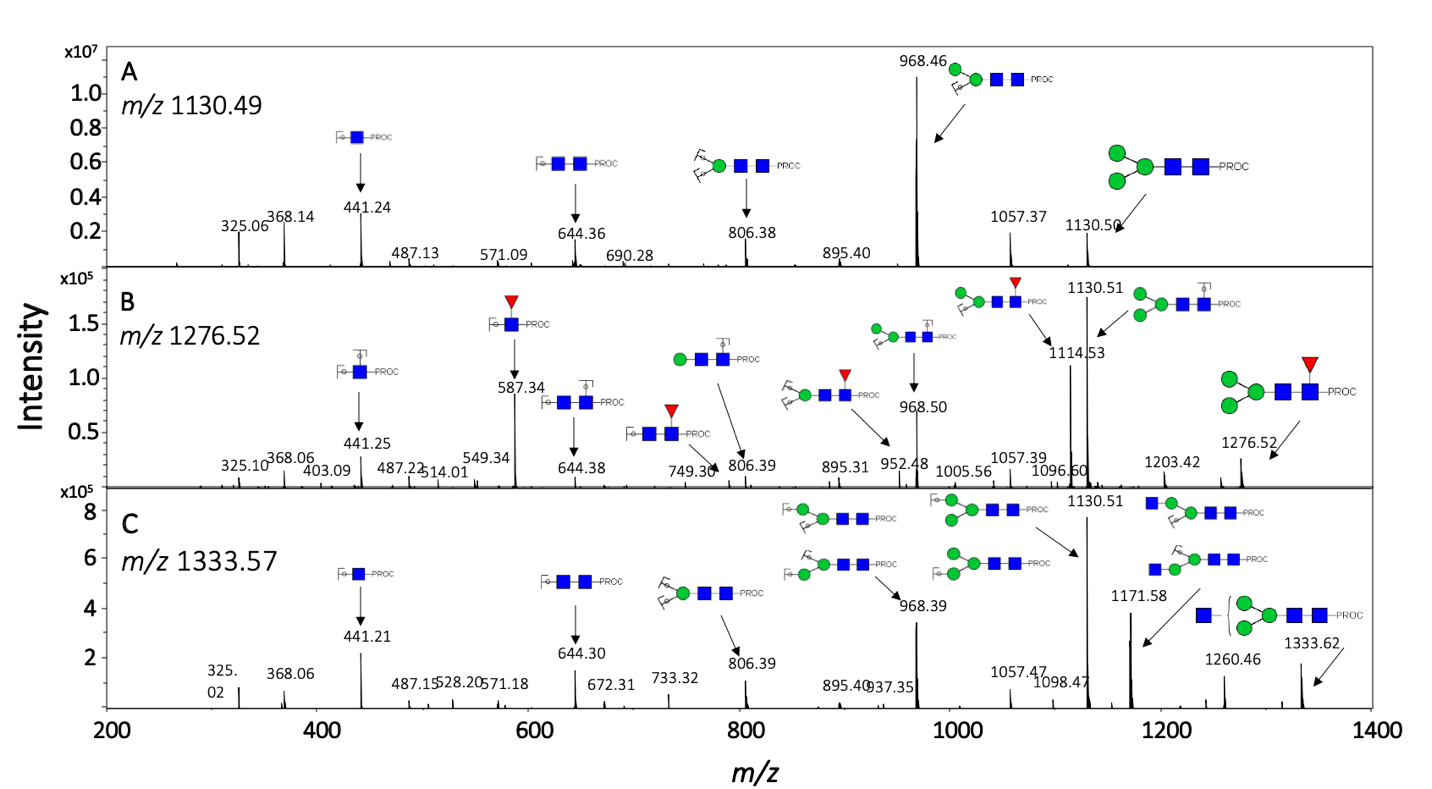


**S4 Fig. Positive-ion ESI-MS/MS fragmentation spectra of procainamide-labelled *N*-glycans from teneral tsetse fly saliva.** Spectra correspond to (A) *m/z* 1130.49 (Man_3_GlcNAc_2_-Proc), (B) *m/z* 1276.52 (Man_3_GlcNAc_2_Fuc-Proc), (C) *m/z* 1333.57 (Man_3_GlcNAc_3_-Proc). Green circle, mannose; blue square, *N*-Acetylglucosamine; red triangle, fucose; Proc, procainamide.
