# Supplementary material for: Tsetse salivary glycoproteins are modified with paucimannosidic N-glycans, are recognised by C-type lectins and bind to trypanosomes
Source: PLoS Negl Trop Dis. 2021 Feb 2;15(2):e0009071. doi: 10.1371/journal.pntd.0009071 (PMC7880456; doi:10.1371/journal.pntd.0009071)
Supplement: S5 Fig — Lane 1, teneral; Lane 2, 4-week old, bloodfed flies (Bloodfed); Lane 3, flies with salivary gland T. brucei infection (Salivary inf.); Lane 4, flies with midgut T. brucei infection (Midgut inf.). (DOCX) [file pntd.0009071.s005.docx]

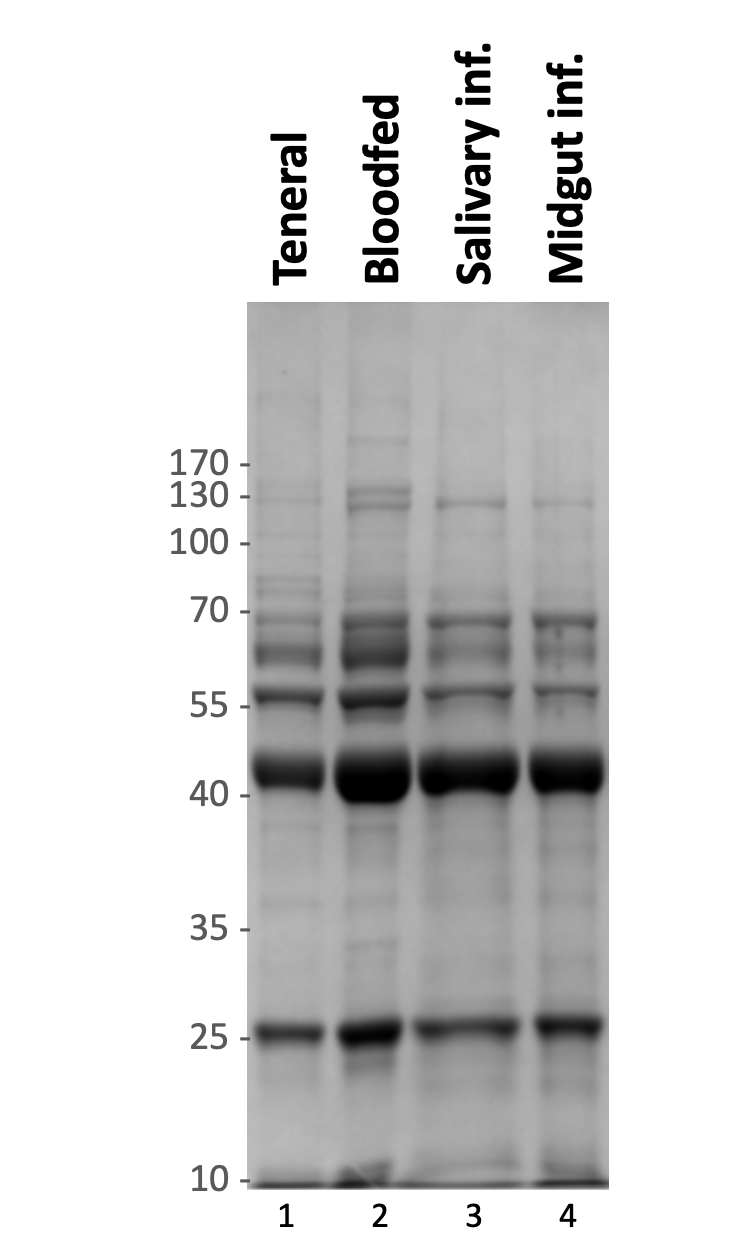


**S5 Fig. SDS-PAGE fractionation of 10 µg of *G. morsitans* salivary profiles obtained from different infection stages**. Lane 1, teneral; Lane 2, 4-week old, bloodfed flies (Bloodfed); Lane 3, flies with salivary gland *T. brucei* infection (Salivary inf.); Lane 4, flies with midgut *T. brucei* infection (Midgut inf.).
