# Supplementary material for: Tsetse salivary glycoproteins are modified with paucimannosidic N-glycans, are recognised by C-type lectins and bind to trypanosomes
Source: PLoS Negl Trop Dis. 2021 Feb 2;15(2):e0009071. doi: 10.1371/journal.pntd.0009071 (PMC7880456; doi:10.1371/journal.pntd.0009071)
Supplement: S6 Fig — (A) Glucose homopolymer (GHP); (B) O-glycans released from bovine fetuin positive control; (C) material released from teneral tsetse saliva. The peaks labelled with asterisks (*) correspond to hydrazinolysis side products (also present in the fetuin and water control samples). (D) water blank (negative control). GU, glucose homopolymer ladder. 2-AB, 2-aminobenzamide. (DOCX) [file pntd.0009071.s006.docx]

**S6 Fig. HILIC-UHPLC profiles of 2-AB labelled material released by hydrazinolysis.** (A) Glucose homopolymer (GHP); (B) *O*-glycans released from bovine fetuin positive control; (C) material released from teneral tsetse saliva. The peaks labelled with asterisks (*) correspond to hydrazinolysis side products (also present in the fetuin and water control samples). (D) water blank (negative control). GU, glucose homopolymer ladder. 2-AB, 2-aminobenzamide.
