# Supplementary material for: Tsetse salivary glycoproteins are modified with paucimannosidic N-glycans, are recognised by C-type lectins and bind to trypanosomes
Source: PLoS Negl Trop Dis. 2021 Feb 2;15(2):e0009071. doi: 10.1371/journal.pntd.0009071 (PMC7880456; doi:10.1371/journal.pntd.0009071)
Supplement: S2 Table — (DOCX) [file pntd.0009071.s008.docx]

**S2 Table. Proteomic identification of tsetse salivary glycoproteins susceptible to PNGase F cleavage (Fig 1).**

| **Band number** | **Apparent molecular mass on SDS-PAGE** | **VectorBase Identifier** | **Name of protein in the band** | **Predicted molecular weight with signal peptide cleaved off** | **Predicted**  ***N*-glycosylation sites (without signal peptide)** | **Unglycosylated sequons detected** | **% Peptide coverage** |
| --- | --- | --- | --- | --- | --- | --- | --- |
| 1 | ~65 kDa | GMOY012313-PA | 5’ Nuc^1^ | 59 kDa | Asn85, Asn173, Asn270, Asn 440 | Asn173 | 45% |
| 2.1 | ~64 kDa | GMOY012372-PA | TSGF2^2^ | 56 kDa | Asn51, Asp103, Asn283, Asn347, Asp484 | Asn484 | 61% |
|  |  | GMOY012375-PA | Adenosine deaminase-related growth factor C^3^ | 54 kDa | Asn19, Asn120, Asn171, Asn370, Asn454, Asn475, Asn483 | Asn120, Asn171, Asn454, Asn475 | 54% |
| 2.2 | ~59 kDa | GMOY012372-PA | TSGF2^2^ | 56 kDa | Asn51, Asp103, Asn283, Asn347, Asp484 | Asn484 | 51% |
|  |  | GMOY012375-PA | Adenosine deaminase-related growth factor C^3^ | 54 kDa | Asn19, Asn120, Asn171, Asn370, Asn454, Asn475, Asn483 | Asn120, Asn171, Asn454, Asn475 | 48% |
| 3.1 | ~57 kDa | GMOY012373-PA | TSGF1^2^ | 54 kDa | Asn339 | - | 59% |
| 3.2 | ~55 kDa | GMOY012373-PA | TSGF1^2^ | 54 kDa | Asn339 | - | 59% |
| 4.1 & 4.2 | ~42 kDa | GMOY012071-PA | Tsal1^4^ | 44 kDa | Asn346 | - | 80% |
|  |  | GMOY012360-PA | Tsal2 (form B)^4^ | 42 kDa | Asn238 | - | 62% |

[1] Caljon *et al*. (2010), [2] Li and Aksoy (2000), [3] Alves-Silva *et al.* (2010), [4] Li *et al*. (2001)

**References**

1. Caljon G, De Ridder K, De Baetselier P, Coosemans M, Van Den Abbeele J. Identification of a tsetse fly salivary protein with dual inhibitory action on human platelet aggregation. PLoS One. 2010;5(3):e9671. Epub 2010/03/31. doi: 10.1371/journal.pone.0009671. PubMed PMID: 20351782; PubMed Central PMCID: PMCPMC2843633.

2. Li S, Aksoy S. A family of genes with growth factor and adenosine deaminase similarity are preferentially expressed in the salivary glands of Glossina m. morsitans. Gene. 2000;252(1-2):83-93. Epub 2000/07/21. doi: 10.1016/s0378-1119(00)00226-2. PubMed PMID: 10903440.

3. Alves-Silva J, Ribeiro JM, Van Den Abbeele J, Attardo G, Hao Z, Haines LR, et al. An insight into the sialome of *Glossina morsitans morsitans*. BMC Genomics. 2010;11:213. Epub 2010/04/01. doi: 10.1186/1471-2164-11-213. PubMed PMID: 20353571; PubMed Central PMCID: PMCPMC2853526.

4. Li S, Kwon J, Aksoy S. Characterization of genes expressed in the salivary glands of the tsetse fly, Glossina morsitans morsitans. Insect Mol Biol. 2001;10(1):69-76. Epub 2001/03/10. doi: 10.1046/j.1365-2583.2001.00240.x. PubMed PMID: 11240638.
