# Supplementary material for: Tsetse salivary glycoproteins are modified with paucimannosidic N-glycans, are recognised by C-type lectins and bind to trypanosomes
Source: PLoS Negl Trop Dis. 2021 Feb 2;15(2):e0009071. doi: 10.1371/journal.pntd.0009071 (PMC7880456; doi:10.1371/journal.pntd.0009071)
Supplement: S4 Table — The symbols for glycan structures are adopted from the Consortium for Functional Glycomics (http://www.functionalglycomics.org/). Red triangle, Fuc; blue square, GlcNAc; green circle, Man; Hex, hexose; HexNAc, N-acetylhexosamine. (DOCX) [file pntd.0009071.s010.docx]

| **S4 Table.** |
| --- |
| HILIC- LC-ESI-MS data with sugar composition and structures for *N*-glycans released by PNGase F and labelled with procainamide. |
| Hex, hexose; HexNAc, *N*-acetylhexosamine |
| The symbols for glycan structures are adopted from the Consortium for Functional Glycomics: |

| **HPLC Peak Id** | **GU (Procainamide)** | **Structure** | **HILIC-LC-ESI-MS** | | | | | | | | | |
| --- | --- | --- | --- | --- | --- | --- | --- | --- | --- | --- | --- | --- |
|  |  |  | **Teneral Fly Saliva N-glycans Procainamide labelled** | | | | | | | | | |
|  |  |  | **Composition** | | | **[*m/z*]^+^ calculated** | **[*m/z*]^2+^ calculated** | **[*m/z*]^+^ registered** | **[*m/z*]^2+^ registered** | **[*m/z*] characteristic fragment ions (composition)** | | |
|  |  |  | **Hex** | **HexNAc** | **Fuc** |  |  |  |  |  |  |  |
| **1** | **3.21** |  | **2** | **2** | **0** | 968.46 | 484.73 | 968.47 | nd | 441.20 (N-PROC) |  |  |
|  |  |  |  |  |  |  |  |  |  | 644.33 (N2-PROC) |  |  |
|  |  |  |  |  |  |  |  |  |  | 806.36 (H1N2-PROC) |  |  |
| **2** | **4.17** |  | **3** | **2** | **0** | 1130.51 | 565.76 | 1130.49 | 565.74 | 441.24 (N-PROC) |  |  |
|  |  |  |  |  |  |  |  |  |  | 644.36 (N2-PROC) |  |  |
|  |  |  |  |  |  |  |  |  |  | 806.38 (H1N2-PROC) |  |  |
|  |  |  |  |  |  |  |  |  |  | 968.46 (H2N2-PROC) |  |  |
| **3** | **4.62** |  | **3** | **2** | **1** | 1276.57 | 638.79 | 1276.52 | 638.77 | 441.25 (N-PROC) | 806.39 (H1N2-PROC) | 1130.51 (H3N2-PROC) |
|  |  |  |  |  |  |  |  |  |  | 587.34 (N1F1-PROC) | 952.48 (H1N2F-PROC) |  |
|  |  |  |  |  |  |  |  |  |  | 644.38 (N2-PROC) | 968.50 (H2N2-PROC) |  |
|  |  |  |  |  |  |  |  |  |  | 790.35 (N2F-PROC) | 1114.53 (H2N2F-PROC) |  |
| **4** | **4.76** |  | **3** | **3** | **0** | 1333.59 | 667.30 | 1333.57 | 667.29 | 441.21 (N-PROC) | 1130.51 (H3N2-PROC) |  |
|  |  |  |  |  |  |  |  |  |  | 644.30 (N2-PROC) | 1171.58 (H2N3-PROC) |  |
|  |  |  |  |  |  |  |  |  |  | 806.39 (H1N2-PROC) |  |  |
|  |  |  |  |  |  |  |  |  |  | 968.39 (H2N2-PROC) |  |  |
| **5** | **5.00** |  | **4** | **2** | **0** | 1292.56 | 646.78 | 1292.54 | 646.74 | 441.24 (N-PROC) | 1130.50 (H3N2-PROC) |  |
|  |  |  |  |  |  |  |  |  |  | 644.32 (N2-PROC) |  |  |
|  |  |  |  |  |  |  |  |  |  | 806.38 (H1N2-PROC) |  |  |
|  |  |  |  |  |  |  |  |  |  | 968.39 (H2N2-PROC) |  |  |
| **6** | **5.54** |  | **4** | **3** | **0** | 1495.64 | 748.32 | 1495.65 | 748.30 | 441.24 (N-PROC) | 1130.58 (H3N2-PROC) |  |
|  |  |  |  |  |  |  |  |  |  | 644.36 (N2-PROC) | 1171.57 (H2N3-PROC) |  |
|  |  |  |  |  |  |  |  |  |  | 806.41 (H1N2-PROC) | 1292.61 (H4N2-PROC) |  |
|  |  |  |  |  |  |  |  |  |  | 968.51 (H2N2-PROC) | 1333.62 (H3N4-PROC) |  |
| **7** | **6.00** |  | **5** | **2** | **0** | 1454.61 | 727.81 | 1454.57 | 727.79 | 441.25 (N-PROC) | 1130.56 (H3N2-PROC) |  |
|  |  |  |  |  |  |  |  |  |  | 644.37 (N2-PROC) | 1292.62 (H4N2-PROC) |  |
|  |  |  |  |  |  |  |  |  |  | 806.38 (H1N2-PROC) |  |  |
|  |  |  |  |  |  |  |  |  |  | 968.44 (H2N2-PROC) |  |  |
|  |  |  |  |  |  |  |  |  |  |  |  |  |
|  |  |  |  |  |  |  |  |  |  |  |  |  |
|  |  | \|  \| \| --- \| |  |  |  |  |  |  |  |  |  |  |
| **8** | **6.46** |  | **5** | **3** | **0** | 1657.69 | 829.35 | 1657.64 | 829.33 | 441.29 (N-PROC) | 1130.55 (H3N2-PROC) | 1454.66 (H5N2-PROC) |
|  |  |  |  |  |  |  |  |  |  | 644.38 (N2-PROC) | 1171.61 (H2N3-PROC) | 1495.73 (H4N3-PROC) |
|  |  |  |  |  |  |  |  |  |  | 806.40 (H1N2-PROC) | 1292.61 (H4N2-PROC) |  |
|  |  |  |  |  |  |  |  |  |  | 968.48 (H2N2-PROC) | 1333.60 (H3N3-PROC) |  |
| **9** | **6.87** |  | **6** | **2** | **0** | 1616.67 | 808.84 | 1616.61 | 808.81 | 441.26 (N-PROC) | 1292.62 (H4N2-PROC) |  |
|  |  |  |  |  |  |  |  |  |  | 644.36 (N2-PROC) | 1454.70 (H5N2-PROC) |  |
|  |  |  |  |  |  |  |  |  |  | 806.39 (H1N2-PROC) |  |  |
|  |  |  |  |  |  |  |  |  |  | 968.49 (H2N2-PROC) |  |  |
|  |  |  |  |  |  |  |  |  |  | 1130.58 (H3N2-PROC) |  |  |
| **10** | **7.79** |  | **7** | **2** | **0** | 1778.72 | 889.86 | 1778.68 | 889.84 | 441.25 (N-PROC) | 1292.62 (H4N2-PROC) |  |
|  |  |  |  |  |  |  |  |  |  | 644.36 (N2-PROC) | 1454.70 (H5N2-PROC) |  |
|  |  |  |  |  |  |  |  |  |  | 806.46 (H1N2-PROC) | 1616.75 (H6N2-PROC) |  |
|  |  |  |  |  |  |  |  |  |  | 968.55 (H2N2-PROC) |  |  |
|  |  |  |  |  |  |  |  |  |  | 1130.55 (H3N2-PROC) |  |  |
| **11** | **8.53** |  | **8** | **2** | **0** | 1940.77 | 970.89 | nd | 970.87 | 441.23 (N-PROC) | 1014.35 (H1N6) | 1500.54 (H1N8) |
|  |  |  |  |  |  |  |  |  |  | 644.29 (N2-PROC) | 1130.45 (H3N2-PROC) |  |
|  |  |  |  |  |  |  |  |  |  | 806.49 (H1N2-PROC) | 1176.41 (H1N5) |  |
|  |  |  |  |  |  |  |  |  |  | 852.27 (H1N4) | 1292.62 (H4N2-PROC) |  |
|  |  |  |  |  |  |  |  |  |  | 968.54 (H2N2-PROC) | 1338.42 (H1N7) |  |
| **12** | **8.66** |  | **8** | **2** | **0** | 1940.77 | 970.89 | nd | 970.87 | 441.24 (N-PROC) | 1014.30 (H1N6) | 1454.59 (H5N2-PROC) |
|  |  |  |  |  |  |  |  |  |  | 644.33 (N2-PROC) | 1130.58 (H3N2-PROC) | 1500.44 (H1N8) |
|  |  |  |  |  |  |  |  |  |  | 806.38 (H1N2-PROC) | 1176.42 (H1N5) |  |
|  |  |  |  |  |  |  |  |  |  | 852.31 (H1N4) | 1292.50 (H4N2-PROC) |  |
|  |  |  |  |  |  |  |  |  |  | 968.44 (H2N2-PROC) | 1338.42 (H1N7) |  |
| **13** | **9.35** |  | **9** | **2** | **0** | 2102.83 | 1051.92 | nd | 1051.90 | 441.28 (N-PROC) | 1014.08 (H1N6) | 1454.51 (H5N2-PROC) |
|  |  |  |  |  |  |  |  |  |  | 644.35 (N2-PROC) | 1130.51 (H3N2-PROC) | 1500.49 (H1N8) |
|  |  |  |  |  |  |  |  |  |  | 806.39 (H1N2-PROC) | 1176.40 (H1N5) | 1662.53 (H1N9) |
|  |  |  |  |  |  |  |  |  |  | 852.25 (H1N4) | 1292.53 (H4N2-PROC) |  |
|  |  |  |  |  |  |  |  |  |  | 970.86 (H2N2-PROC) | 1338.38 (H1N7) |  |
