# Supplementary material for: Tsetse salivary glycoproteins are modified with paucimannosidic N-glycans, are recognised by C-type lectins and bind to trypanosomes
Source: PLoS Negl Trop Dis. 2021 Feb 2;15(2):e0009071. doi: 10.1371/journal.pntd.0009071 (PMC7880456; doi:10.1371/journal.pntd.0009071)
Supplement: S5 Table — Table shows details for three representative glycan structures from saliva from teneral flies. *Mass corresponds to loss of diethylamine ion (73 Da). (DOCX) [file pntd.0009071.s011.docx]

| **S5 Table.** | | | | | | | | | | |  |  |
| --- | --- | --- | --- | --- | --- | --- | --- | --- | --- | --- | --- | --- |
| \| HILIC-LC-ESI-MS/MS data for *N*-glycans released by PNGase F. Table shows details for three representative glycan structures from saliva from teneral flies. \| \| --- \| \| *Mass corresponds to loss of diethylamine ion (73 Da) [1]. \| | | | | | | | | | | |  |  |
| **HILIC Peak ID** | **Structure** | **Composition** | | | **[*m/z*]^+^ calculated** | **[*m/z*]^+^ registered** | **[*m/z*] characteristic fragment ions (composition)** | | | | | |
|  |  |  |  |  |  |  |  |  |  |  |  |  |
| 2 |  | **Hex** | **HexNAc** | **Fuc** | 1130.51 | 1130.49 | 325.06 (H2) | 571.09 (N2-PROC)* | 806.38 (H1N2-PROC) | |  |  |
|  |  | 3 | 2 | 0 |  |  | 368.14 (N-PROC)* | 644.36 (N2-PROC) | 895.40 (H2N2-PROC)* | |  |  |
|  |  |  |  |  |  |  | 441.24 (N-PROC) | 733.14 (H1N1-PROC)* | 968.46 (H2N2-PROC) | |  |  |
| 3 |  | 3 | 2 | 1 | 1276.57 | 1276.52 | 325.10(H2) | 571.22 (N2-PROC)* | 790.35 (N2F-PROC) | | 968.50 (H2N2-PROC) | 1130.51 (H3N2-PROC) |
|  |  |  |  |  |  |  | 368.06 (N-PROC)* | 587.34 (N1F1-PROC) | 806.39 (H1N2-PROC) | | 1041.41 (H2N2F-PROC)* |  |
|  |  |  |  |  |  |  | 441.25 (N-PROC) | 644.38 (N2-PROC) | 895.31 (H2N2-PROC)* | | 1057.39 (H3N2-PROC)* |  |
|  |  |  |  |  |  |  | 514.00 (N1F1-PROC)* | 733.09 (H1N2-PROC)* | 952.48 (H1N2F-PROC) | | 1114.53 (H2N2F-PROC) |  |
| 4 |  | 3 | 3 | 0 | 1333.59 | 1333.57 | 325.02 (H2) | 644.30 (N2-PROC) | 968.39 (H2N2-PROC) | | 1260.46 (H3N3-PROC)* |  |
|  |  |  |  |  |  |  | 368.06 (N-PROC)* | 733.32 (H1N2-PROC)* | 1057.47 (H3N2-PROC)* | | 1171.58 (H2N3-PROC) |  |
|  |  |  |  |  |  |  | 441.21 (N-PROC) | 806.39 (H1N2-PROC) | 1098.47 (H2N3-PROC)* | |  |  |
|  |  |  |  |  |  |  | 571.18 (N2-PROC)* | 895.40 (H2N2-PROC)* | 1130.51 (H3N2-PROC) | |  |  |

[1] Kozak, R. P., Tortosa, C. B., Fernandes, D. L. and Spencer, D. I. (2015) Comparison of procainamide and 2-aminobenzamide labeling for profiling and identification of glycans by liquid chromatography with fluorescence detection coupled to electrospray ionization-mass spectrometry. Anal Biochem. 486, 38-40
