# Supplementary material for: Tsetse salivary glycoproteins are modified with paucimannosidic N-glycans, are recognised by C-type lectins and bind to trypanosomes
Source: PLoS Negl Trop Dis. 2021 Feb 2;15(2):e0009071. doi: 10.1371/journal.pntd.0009071 (PMC7880456; doi:10.1371/journal.pntd.0009071)
Supplement: Attachment — Submitted filename: Response to reviewers.docx [file pntd.0009071.s012.docx]

**Responses to reviewers**

We would like to thank the reviewers for positive comments and suggestions. Please find below the responses (in red) to each point; we hope we have addressed them satisfactorily.

- Reviewer #2: The results from the structural determinations are conclusive and, although no significant differences between N-glycosylation of infected and non-infective salivary proteins were found, the knowledge of this fact contribute to the understanding of host-parasite interaction. Biological tests are more preliminary and encourage further studies on the subject. Particularly, since glycans seem to be essential to the binding of the protein to the parasite it would be interesting to determine which structures are present in the most abundant bound protein.
- Reviewer #3: The conclusions are supported by the data, although limitations of the N-glycosylation analysis performed should be stated in the discussion. The data presented is very valuable, but it is not a site-specific analysis, which could be a second step in the future for a thorough characterization of the N-glycoproteins described here, and could help understand the differences in the results pertaining lectin recognition.

The following has been added to the Discussion: ‘further characterization of the *Glossina* N-glycome should include site-specific analyses in order to better understand the influence of glycosylation in the role of the salivary protein.’ (Line 516-518).

- Moreover, and considering that data on O-glycosylation is not shown, all the manuscript should be thoroughly revised and corrected in order not to induce the reader on the misconcept that the salivary glycome of the Tse Tse fly is characterized. The text reads “glycome, glycans, glycosylation”, but the study is limited to the N-glycome, N-glycans, and N-glycosylation. Even though the authors do mention that they perform an O-glycan analysis by HILIC or MS with negative results, the data is not shown and only mentioned in the Discussion.
  We have modified the mentions of glycome, glycans and glycosylation, were relevant, to specify that these are N-linked glycans we are talking about throughout the manuscript.

Additionally, we have included in the Discussion a comment on the search for *O*-glycans in tsetse saliva (Line 586-588), included a brief method paragraph (lines 235-239) and a new figure (S6 Fig) as part of the Supplementary information section.

- Reviewer #1: Please cite figure 3B in the text

Figure 3 is now Figure 4. Figure 4B is the nigrosine-stained (loading) control for experiment shown in 4A as indicated in the legend (line 443-446); they are both cited in line 623. Furthermore, we have included citation for Figure 4C (Line 440).

- Figure 4, indicate the salivary proteins in the metacyclic trypanosome lysate with an asterisks as stated in the figure legend.

We thank the reviewer for spotting this – we have added the asterisks to the figure (now Figure 5).

- Reviewer #2: In the conclusions the authors stated that they did not found O-glycosylation. Even though it is a no shown result, it should be mention how this data was obtained. That is, if the glycoproteins were treated chemically or enzymatically to release the O-linked glycans.
  As indicated above, manuscript has been updated with “To evaluate the presence of *O*-linked glycans in tsetse salivary glycoproteins, we also released the *O*-glycans by hydrazinolysis [58] and analyzed fluorescently labelled samples by HILIC-HPLC and MS. However, we did not detect *O*-glycans by either HILIC or MS (S6 Fig)” (Line 586-588), a new method paragraph (lines 235-239) and a new figure (S6 Fig).
- Reviewer #3: a) General:
  All text should be corrected in order not to induce the reader on the misconcept that the salivary glycome of the Tse Tse fly is characterized. The text reads “glycome, glycans, glycosylation” in many sentences and sections, and the study is limited to the N-glycome, N-glycans, and N-glycosylation. Even though the authors do mention that they did not detect O-glycans, the data is not shown and only mentioned in the Discussion (Line 543). I believe that the manuscript should be more specific on the specific data on N-glycosylation provided as results.
  As requested:

- We have modified the mentions of glycome, glycans and glycosylation, were relevant, to specify that these are N-linked glycans we are talking about throughout the manuscript.

- As indicated above, manuscript has been updated regarding the attempt to find *O*-glycans in tsetse saliva Line 586-588), a brief method paragraph a new figure (S6 Fig).

- b) Materials and Methods
  Line 249: Streptococcus pneumonia should read Streptococcus pneumoniae

Thanks for the correction. Species name has been corrected (line 256).

- Line 250: Jack Bean Mannosidase is abbreviated as JBM in all the text, apart from this section, where it is defined as JBAM. For the sake of clarity, I suggest homogenizing this with the rest of the text.

As requested, all instances have been homogenized to JBM throughout the manuscript.

- In this section, and considering that bkF and JBM are not compatible in their assay requirements, a brief description on the sequential digestion bkF+JBM should be added.

Manuscript updated with “and Jack bean α-(1-2,3,6)-Mannosidase (JBM, QA-Bio). The bkf enzyme contains approximately ≤0.2% β-N-acetylglucosaminidase and ≤0.1% of α-mannosidase and β-galactosidase, thus combination of bkf and JBM is necessary to achieve full and final sequencing” (Line 257-260). For combined digestions/exo arrays we used 500 mM sodium acetate buffer pH 5.5.

- Line 255: title should read HILIC-UHPLC-FLR, as N-glycans are specifically detected by tagging with a fluorescent probe.

Section title has been completed to reflect the method (line 264).

- Results:
  Section: Identification of tsetse salivary glycoproteins
  I consider that Supplementary Figures 1-3 associated to this section should be incorporated in the main manuscript. Experimental data on N-glycosylated proteins in saliva of Glossina morsitans is rather scarce and is mostly based on predictions, so I think this part of the paper should be incorporated as a Figure.

Agree – Supplementary figure S2 has been changed to Figure 1, and we have updated all figure numbering accordingly.

- Line 317: “Taken together, these results indicate the presence of few key glycoproteins in tsetse fly saliva, and more importantly, the presence of several high mannose or hybrid type N-glycans.” Authors should consider the possibility of lower abundance N-glycosylated proteins that were not detected here as by technical limitations. The final statement should be restricted to those glycoproteins characterized.

The sentence has been modified as follows (Line 331-333): ‘These comprise the most abundant glycoproteins in the saliva of the tsetse fly; however, we cannot discount the existence of proteins in very low abundance that we could not detect by our methods.’

- Line 323: As it is referring to 2AB-labelled N-glycans, it should read HILIC-UHPLC-FLR. Also in the legend Figure 1, reads HILIC-(U)HPLC, please homogenize abbreviations.

Line 264 and line 338: -FLR has been added.

Line 352: Figure 1 (now Figure 2) legend has been corrected (parenthesis removed in ‘HILIC-UHPLC’).

- Figure 1: In the two panels the authors use an asterisk to mark different issues. In Panel A, they highlight buffer contaminants. In Panel B, they mark the m/z 1130.55 as [M+2H]2+ ion, which corresponds to the most abundant N-glycan (Man3GlcNAc2), which has two ions associated to it in this panel. I would advice to use different ways to highlight these issues, as they are very different and it can be confusing to the reader.

Figure 1 is now Figure 2. As suggested, we have changed the symbol in panel B (now ‡) to differentiate this note from the asterisk in panel A.

- Considering that the peak at around 5 minutes RT in Figure 1A appears with different intensities in other chromatograms, it should be marked in all of them.

Thanks for spotting this. Peaks corresponding to contaminants have been marked in all chromatograms.

- Moreover, and regarding the structure Man3GlcNAc2, I believe a short sentence explaining why this particular structure appears as a double peak could help the MS non-specialist reader understand the reasons behind this. In the Results describing Figure 1B this peak is not discussed and only appears in the description on the fragmentation data (Supplementary Figure S5).

The following has been added to the legend in Figure 2:

“the appearance of the Man_3_GlcNAc_2_-Proc as singly and doubly charged ion in positive mode, reflects on its high relative abundancy (~54%) in this sample” (Line 356-357).

- A brief explanation for the CFG glycan symbology is required in the legend for this figure, it is only presented in Supplementary Table 4, and without a reference, which should be added for non-specialized readers.

Figure 2 legend already ends with this sentence, which explains the glycan symbology used in the figure: Green circle, mannose; blue square, *N*-Acetylglucosamine; red triangle, fucose; Proc, procainamide.

- The chromatograms are shown with retention time as an X axis, and Table 1 describe GU values. There is no description or reference in Materials and Methods to the usage of Dextran as external standard or how GUs are calculated. GUs are not described in the Figure. This can be thoroughly confusing for a non-specialized reader, considering the broad scope of this Journal.

This information is already in the manuscript under Materials and Methods – p12, Line 274. In addition, GU was added to: (i) Fig 2, (ii) Fig 2 legend, and (iii) Table 1.

- Peak 13 (Man9GlcNAc2) described in 1A is missing in 1B, no explanation for this.
  Figure 2 has been updated to include a complete version with peak 13.
- Table 1: Exoglycosidase digestion of 2-AB-labelled glycans released from teneral fly saliva by PNGase F. The first column should read HILIC-UHPLC, as retention times can be very different in HPLC.

Table has been updated as mentioned above.

- I think it would really improve the presentation of data if authors could add a new column with the MS data from Figure 1B, so the reader can integrate the information. I understand MS analysis is based on procainamide-labelled N-glycans, but this can be specified in the Table.

Thanks for your suggestion. We believe however that this could be hard to follow and perhaps unnecessary given that, for example, after JBM digestion most glycans will result in the formation of Man_1_GlcNAc_2_ (peak A – Figure 2A); additionally, all the MS data can be found in the Supplementary Table.

- In Figure 1A, and to complement this table, I would indicate with arrows the shifts in RT caused by digestions.

We believe that this is not really necessary for esthetical reasons and clarity – it would make figure 1A (now Figure 2A) too busy/saturated.

- Finally, I would also add a column before the structure column with the abbreviation used in the text for each glycan (the text uses abbreviations such as Man3GlcNAc2 with few references to the peak numbers, and in the table only the CFG symbology and peak number is used).
  We agree with your suggestion. However, for simplicity, the abbreviation for each glycan has been now added underneath the corresponding structure (see Table 1).
- Line 381 and following paragraph: “We then determined whether T. b. brucei infection alters the structure of salivary N-glycans. Oligosaccharides were released with PNGase F, labelled with procainamide, and then analyzed by HILIC coupled with ESI-MS. Figure 2A shows the HILIC chromatogram, where no change is observed in the saliva N-glycan population following infection of the salivary gland. A comparison of the relative percentage areas of glycan peaks identified in teneral, naïve and infected fly saliva (Table 2) showed no quantitative differences after infection either.”

In Figure 2, chromatograms look exactly the same as those in Figure 1, and corresponding legend states that the chromatograms are from 2AB-labelled glycans. The text should be corrected, as from this paragraph, it seems that chromatograms are based on procainamide labelling, not 2AB.

Thanks for spotting this. Information has been amended as follow:

Figure 3A - The HILIC-UHPLC chromatograms and data (Table 2) is for 2-AB labelled samples.

Figure 3B – ESI-MS/MS data is for procainamide-labelled samples.

- Peak 13 is again missing in 2B. If it cannot be seen in the MS due to technical issues (i.e. low ionization), please indicate so, as it is also missing in Figure 1B.
  Thanks for noticing this. Figure 2 has been updated to include a complete version with peak 13.
- The description of the “no differences” in N-glycosylation following infection is quite confusing.
  “A comparison of the relative percentage areas of glycan peaks identified in teneral, naïve and infected fly saliva (Table 2) showed no quantitative differences after infection either.”
  There are differences between teneral and naïve N-glycans, there are no differences between infected and naïve samples. Analysis should be rephrased by pairing of comparable samples (teneral vs naïve, naïve vs bloodfed).
  “This is confirmed by the MS spectra (Figure 2B) which shows that salivary glycan structures and abundances remain unaffected by infection with T. brucei. However, when comparing teneral versus naïve (bloodfed) flies or infected flies, there seemed to be a slight variation in the abundance of some structures (Table 2: peaks 4, 9, 10, 13), potentially an effect of blood ingestion."
  Just to follow up on the previous comments, peaks in this sentence are only defined by number, and not by name. Moreover, how can differences/similarities in abundance be quantified from Figure 2B? Have the authors compared intensities for procainamide labelled N-glycans obtained from infected and naïve samples using the BPI data channel? That is not shown in Figure 2B.
  We agree with the observation. The text has been updated to state “A comparison of the relative percentage areas of glycan peaks identified in teneral, naïve and infected fly saliva (Table 2) showed a slight variation in the abundance of some structures (Table 2: peaks 4, 9, 10, 13), potentially an effect of blood ingestion.” (Line 415-417).
- Table 2: Comparison of relative abundance of released N-glycans present in saliva samples from teneral (unfed), naïve and T. brucei-infected flies.
  As per materials and methods, it should read HILIC-UHPLC instead of HPLC. Here again GU values are used and not described. Moreover, it should clarify that it was done with 2AB.

As mentioned previously, Table has been updated, and “GU, glucose units” was added to the table legend. In addition, the following has been added to the legend: “Data correspond to 2-AB labelled samples.”

- Line 385: Teneral and naïve should be defined as this is the first part of the results where they are used. They are generally defined in Materials and Methods, Figure Legends and Tables, but it would be really useful to have it defined in the Results as first time mentioned.
  The terms teneral and naïve have been introduced in the Results section (Line 397-398).
- Discussion:
  Line 457: "Through enzymatic analysis coupled with highly sensitive LC and ESI-MS/MS, we found that salivary N-glycans in G. morsitans saliva are mainly represented by the paucimannose Man3GlcNAc2 structure, with the addition of a few hybrid type oligosaccharides."
  This sentence should be corrected as high mannose N-glycans are also detected.

We have added high mannose N-glycans to the sentence (line 498).

- Line 468: When comparing to data on other insects, absence of a(1,3) fucosylated N-glycans in G. morsitans (shown by the authors by digestion with PNGase A) should be present in the discussion.
  We have added the following to the text “however, we did not find evidence of α1-3 linked fucosylation in the saliva of this fly” (line 506-507).
- Line 470: are sgp3 and Gmmsgp3 the same? If so, please homogenize the abbreviation.
  The name of this salivary protein has been homogenized to Gmmsgp3 throughout the manuscript.
- Line 502 and corresponding paragraph: “We also show here that a recombinant fraction of the DC-SIGN recognizes some salivary glycoproteins from tsetse saliva. Notably, the DC-SIGN seems to recognize fewer tsetse glycoproteins than the mannose receptor, possibly due to its specificity for high-mannose glycan structures [43], which would not recognize the most abundant Man3GlcNAc glycan species.”

Even though I understand this is a first approach to interactions with soluble forms of MR and DC-SIGN, a correlation with the N-glycoproteins identified in the previous sections of the manuscript could be provided, in order to integrate this data with all previous work in the manuscript.
We agree with your suggestion. However, the *G. morsitans* sialome (protein composition) is comprised of hundreds of proteins, so a correlation on the possible protein identified by lectin blotting alone would be inaccurate. A lectin chromatography/MS approach would be needed in order to identify the protein(s) that are poorly recognised by this lectin.

- Data published in the following paper could also be relevant to the discussion of the results:
  Ellis JA, Shapiro SZ, ole Moi-Yoi O, Moloo SK. Lesions and saliva-specific antibody responses in rabbits with immediate and delayed hypersensitivity reactions to the bites of Glossina morsitans centralis. Vet Pathol. 1986 Nov;23(6):661-7. doi: 10.1177/030098588602300603. PMID: 3811131.
  Thanks for the suggestion. This reference (listed as [36]) has been added to the manuscript.

Finally, and as previously stated, the discussion should consider the limitations of de-N-glycosylation in identifying specific structures for each glycoprotein and N-glycosylation site, something that can be achieved by site-specific analysis.
As stated above, the following has been added to the Discussion: ‘further characterization of the *Glossina* N-glycome should include site-specific analyses in order to better understand the influence of glycosylation in the role of the salivary protein.’ (Line 516-518).

- Supplementary data:
  Title in supplementary data does not correspond to the title in the Manuscript.
  We have corrected the title of the supplementary material
- Supplementary Tables 1 and 2: It would be easier to follow the results if authors could indicate the same band numbers in Figures and Tables.

Supplementary Tables 1 and 2 are for different datasets. Suppl. Table 1 summarises the *in silico* search for N-glycosylation sequons in *Glossina morsitans* salivary proteins. In Suppl. Table 2, band numbers correspond to those observed in the new Figure 1; as expected from a MS proteomics analysis, in some bands more than one protein was identified, which is why they are numbered for instance 2.1 and 2.2 (two salivary proteins corresponding to band 2).

- The reference “Li and Aksoy, 2000” is not cited in the References. Is this one?
  Song Li, Serap Aksoy, A family of genes with growth factor and adenosine deaminase similarity are preferentially expressed in the salivary glands of Glossina m. morsitans, Gene, Volume 252, Issues 1–2, 2000, Pages 83-93, ISSN 0378-1119, <https://doi.org/10.1016/S0378-1119(00)00226-2>
  The reference “Li et al., 2001” is not cited in references. Is this one?
  Li S, Kwon J, Aksoy S. Characterization of genes expressed in the salivary glands of the tsetse fly, Glossina morsitans morsitans. Insect Mol Biol. 2001 Feb;10(1):69-76. doi: 10.1046/j.1365-2583.2001.00240.x. PMID: 11240638.

Apologies for the oversight. Both of these references have been added in a complete format to supplementary table 2, as well as to the main manuscript and reference list.

- Apart from these two missing references, Ref. 1 and 3 in Supplementary Data are Ref. 33 and 17, respectively, in the main text.
  All references in Supplementary Data should be included and correlatively numbered with the main reference list. If not present there, and if the Journal allows it, they could also be added a list of supplementary references.

All references have been completed where necessary.

- Figure S4: For better comparison, structures or GU values should be added.

Structures have been added to the figure.

- Peak around 5 min should be marked as a contaminant.

Corresponding peaks have been marked as contaminants with an asterisk.

- Legend reads: "Figure S4. HILIC-(U)HPLC chromatograms of 2-AB labelled N-glycans released enzymatically from teneral fly saliva. (A) N-glycans released by PNGase A, (B) N-glycans released by PNGase F, (C) Nglycans released by PNGase A after deglycosylation with PNGase F. The glycan structures corresponding to the numbers on the HPLC peaks are listed in Table 3."
  There is no Table 3 (corresponds to Table 1?), and the chromatography corresponds to HILIC-UPLC-FLR, not HPLC.
  Apologies for the mistake. Figure legend has been edited and now reads: “**S3 Fig.** HILIC-UHPLC chromatograms of 2-AB labelled *N*-glycans released enzymatically from teneral fly saliva. (A) *N*-glycans released by PNGase A, (B) *N*-glycans released by PNGase F, (C) *N*-glycans released by PNGase A after deglycosylation with PNGase F. Green circle, mannose; blue square, *N*-acetylglucosamine; red triangle, fucose. Peaks labelled with an asterisk refer to buffer contaminants.” (line 837-840).
